# INCREASING DEMENTIA DETECTION IN HEALTH CARE SYSTEMS: LEARNINGS FROM THREE PRAGMATIC CLINICAL TRIALS

**DOI:** 10.1093/geroni/igae098.1926

**Published:** 2024-12-31

**Authors:** Sascha Dublin, Deborah Barnes, Shari Ling

**Affiliations:** Chair; Co-Chair; Discussant

## Abstract

In the US, an estimated 6.7 million older adults have dementia, yet only about half have received a diagnosis. Earlier detection has potential to improve health and quality of life for people living with dementia and their caregivers by improving access to education, supportive services, and emerging treatments. The National Institute on Aging has funded pragmatic clinical trials of innovative, low-cost approaches aimed at increasing recognition of dementia within healthcare systems. In this symposium, we will present methods and early results from 3 such trials. The eRADAR study is applying a machine learning-based algorithm that draws on electronic health records (EHR) data to identify high-risk individuals. The intervention involves inviting high-risk individuals in the intervention arm for a “brain health” assessment visit within primary care. The D Cubed Study is testing two approaches: an EHR-based algorithm, the Passive Digital Marker, and a brief patient-reported questionnaire, alone and in combination. Results are shared with primary care providers via the EHR together with next steps for evaluation. The Toolbox Detect study is examining whether self-administered, brief cognitive screening tests given via tablet computers as part of annual wellness visits can increase dementia detection. Investigators leading these trials will describe their interventions and study methods and present early results related to uptake, feasibility and acceptability. They will also discuss challenges encountered, potential solutions, and lessons learned. A major goal will be to engage audience members to consider how they might apply these methods to increase detection of dementia in their own settings.

